# The enduring advantages of the SLOW5 file format for raw nanopore sequencing data

**DOI:** 10.1093/gigascience/giaf118

**Published:** 2025-10-16

**Authors:** Hasindu Gamaarachchi, Sasha Jenner, Hiruna Samarakoon, James M Ferguson, Ira W Deveson

**Affiliations:** School of Computer Science and Engineering, University of New South Wales, Sydney, NSW, 2052, Australia; Genomics and Inherited Disease Program, Garvan Institute of Medical Research, Sydney, NSW, 2010, Australia; Genomics and Inherited Disease Program, Garvan Institute of Medical Research, Sydney, NSW, 2010, Australia; School of Computer Science and Engineering, University of New South Wales, Sydney, NSW, 2052, Australia; Genomics and Inherited Disease Program, Garvan Institute of Medical Research, Sydney, NSW, 2010, Australia; Genomics and Inherited Disease Program, Garvan Institute of Medical Research, Sydney, NSW, 2010, Australia; Genomics and Inherited Disease Program, Garvan Institute of Medical Research, Sydney, NSW, 2010, Australia; St Vincent’s Clinical School, Faculty of Medicine, University of New South Wales, Sydney, NSW, 2011, Australia

**Keywords:** nanopore, raw signal, SLOW5, POD5, file formats, sequencing data, genomics data

## Abstract

Nanopore sequencing is a widespread and important method in genomics science. The raw electrical current signal data from a typical nanopore sequencing experiment are large and complex. This can be stored in 2 alternative file formats that are presently supported: POD5 is a signal data file format used by default on instruments from Oxford Nanopore Technologies (ONT); SLOW5 is an open-source file format originally developed as an alternative to ONT’s previous file format, which was known as FAST5. The choice of format may have important implications for the cost, speed, and simplicity of nanopore signal data analysis, management, and storage. To inform this choice, we present a comparative evaluation of POD5 versus SLOW5. We conducted benchmarking experiments assessing file size, analysis performance, and usability on a variety of different computer architectures. Binary SLOW5 (BLOW5) showed superior performance during sequential and nonsequential (random access) file reading on most systems, manifesting in faster, cheaper basecalling and other analysis, and we could find no instance in which POD5 file reading was significantly faster than BLOW5. We demonstrate that BLOW5 file writing is highly parallelizable, thereby meeting the demands of data acquisition on ONT instruments. Our analysis also identified differences in the complexity and stability of the software libraries for SLOW5 (slow5lib) and POD5 (pod5), including a large discrepancy in the number of underlying software dependencies, which may complicate the pod5 compilation process. In summary, many of the advantages originally conceived for SLOW5 remain relevant today, despite the replacement of FAST5 with POD5 as ONT’s core file format.

## Introduction

Nanopore sequencing is widely used for genome, epigenome, and transcriptome analysis [1], and nanopore-based protein sequencing is in development [2]. Its leadership in nanopore development and applications has seen Oxford Nanopore Technologies (ONT) grow into one of the world’s leading genomics companies.

ONT instruments measure the displacement of ionic current as a DNA or RNA molecule passes through a nanoscale protein pore. The resulting time-series current signal data can be translated or “basecalled” into DNA/RNA sequence reads. Raw ONT signal data may also be analyzed directly to identify modified DNA or RNA bases [3–5], DNA damage [6], RNA secondary structures [7, 8], or for a variety of other purposes [9, 10]. Because basecalling models and other analysis algorithms are rapidly evolving, the retention and reanalysis of raw ONT signal data is a common practice.

We previously developed a nanopore signal data file format called SLOW5 (and its binary equivalent BLOW5) [11]. SLOW5 was conceived as an open-source alternative to ONT’s standard data format at the time, called FAST5. SLOW5 addressed limitations in FAST5 and its underlying software library HDF5, which prevented parallel access by multiple CPUs, enabling SLOW5 to achieve order-of-magnitude acceleration of signal data analysis and 20–50% reductions in file size [11]. Such performance enhancements may have profound benefits for users, dramatically reducing the time and costs associated with nanopore data analysis, management, and storage. SLOW5 was also a simpler format than FAST5, making it easier both for developers and users to interact with raw ONT signal data [11].

ONT later developed a new signal data file format to replace FAST5, called POD5 [12]. POD5 was designed to fix the design flaws identified in FAST5, enabling faster analysis and smaller file sizes. POD5 is now the signal data file format used by default on ONT sequencing instruments, marking it as a mature format that is suitable for evaluation.

The replacement of FAST5 with POD5 has undoubtedly enhanced ONT as a platform and benefited ONT users. However, SLOW5 was a mature, open-source format available for adoption by ONT prior to the development of POD5. Here we address the question of whether the decision to develop POD5, rather than adopt SLOW5, has resulted in a superior core file format for ONT sequencing. We compare the POD5 and SLOW5 formats, as well as present benchmarking experiments evaluating file size, analysis performance, and usability on a diverse range of computer architectures. Our comparison of POD5 versus SLOW5 is intended to inform nanopore users, developers, and commercial vendors on the optimal choice of file format for nanopore signal data.

## Results

### Comparison of POD5 versus SLOW5 file formats

The POD5 and SLOW5 file formats contain an equivalent set of metadata and identical raw signal data. Bidirectional lossless conversion between the 2 formats is therefore possible. Conversion may be performed using the software package blue-crab [13].

**Figure 1:**
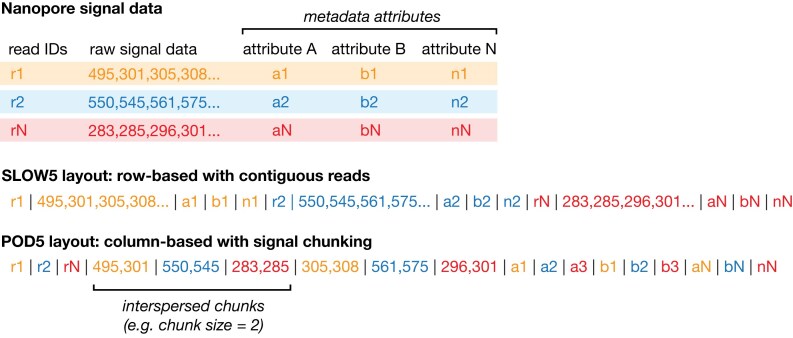
Comparison of SLOW5 versus POD5 file layout. Schematic shows a simplified representation of the layout used to store nanopore signal data in SLOW5/BLOW5 versus POD5 file formats. Upper section shows the basic elements making up a raw nanopore read (3 reads shown in different colors): a unique read identifier, a set of metadata attributes describing the read (e.g., channel number, read number, mux values), and the data values for that read. Lower section shows how these elements are serialized onto a linear computer file in SLOW5 format and POD5 format, respectively. SLOW5 uses a row-based layout with all elements for a single read stored contiguously. POD5 uses a column-based layout and additionally stores raw data in “chunks,” with chunks from multiple reads allowed to be interspersed within the file. A chunk size of 2 is used here for illustrative purposes, meaning each chunk contains 2 signal data values (the current default chunk size in POD5 files is 102,400).

The most obvious difference between the formats is their serialization or layout. Like most other common genomics file formats (e.g., SAM, FASTA, VCF, BED), SLOW5 is a row-based format. The metadata and all signal data from a given read are stored contiguously, and independent reads are laid on a SLOW5 file in sequential rows (Fig. 1; Table 1). In contrast, POD5 is column-oriented, with each metadata attribute stored for all reads, followed by signal data for all reads (Fig. 1; Table 1). In a SLOW5 file, all signal data points for a given read are adjacent, whereas POD5 stores signal data in subreads or “chunks” of a fixed length, and chunks from multiple reads may be interspersed (Fig. 1). This layout facilitates writing of multiple reads in parallel, directly to the disk, which is advantageous during data acquisition. However, it means multiple seek operations are required to access all signal chunks and metadata attributes for a given read within the file during data analysis.

**Table 1:** Comparison of SLOW5 versus POD5 file formats for nanopore signal data

Feature	S/BLOW5	POD5
Layout	Row-based	Column-based
Underlying format	None; SLOW5/BLOW5 format written from scratch	Apache Arrow Interprocess Communication (IPC) format; also known as Apache Feather v2 format
Variants	ASCII SLOW5 for human readability; binary BLOW5 for computer readability	Binary variant only
Signal contiguity	Signal data for each read stored contiguously	Signal data stored in chunks; chunks from a given read may be adjacent or interspersed with chunks from other reads
Primary I/O method	Traditional buffered I/O	Memory-mapped (mmap) I/O
Accessing a given set of reads	Index file-based	No index file; reads accessed by a “walker” that traverses through the file

SLOW5 is a tab-delimited ASCII format with a direct binary equivalent, BLOW5, that does not depend on any other underlying format. POD5 is itself a layer built on top of a primary file format called Apache Arrow Interprocess Communication (IPC) format (Table 1). SLOW5/BLOW5 files are read and written by an accompanying library, called slow5lib, which accesses the files through standard C library functions that are very close to the operating system/hardware. POD5 files are read and written by an accompanying library, called pod5, which is built on the Apache Arrow library, which is itself dependent on the library C++17 (Table 1). Secondary software libraries for reading/writing SLOW5/BLOW5 and POD5 files are also available in other languages, such as Python and R.

### Benchmarking analysis overview

To evaluate SLOW5 and POD5 formats, we conducted benchmark experiments assessing their basic properties, including file sizes and performance during file reading and writing. We further examined how differences in these properties manifest during real-world analysis scenarios, such as ONT basecalling. We have previously demonstrated that the choice of file format has no impact on analysis outcomes [11, 14]. Therefore, we focus here exclusively on performance metrics such as time and memory use, taking equivalent outputs as a given. The binary form of SLOW5 (BLOW5) was used for all experiments. The primary C/C++ libraries for each format were used during all benchmark experiments, and secondary libraries in Python, R, Rust, or other languages were not considered. Because performance is influenced by the architecture of the computer on which the data are stored and the analysis is executed, our benchmark experiments are conducted on an array of different systems, ranging from commercial cloud computing platforms to laptops and a smartphone (Table 2). Experiments were run using all available CPU threads on a given system, unless otherwise specified. Finally, we conducted a semi-quantitative assessment of “usability,” considering factors such as code complexity, software dependencies, and backward/forward compatibility for each file format and its primary software library.

**Table 2:** Overview of computer environments used during benchmarking experiments

System name	Description	n × CPU	Total cores, CPU threads	RAM (GB)	Disk system and file system	O/S
hpc-ncigadi-cpu	NCI CPU server node	2 × Intel Xeon Platinum 8274	48, 92	188	Lustre distributed file system	Rocky Linux 8.10
hpc-ncigadi-gpu	NCI GPU server node with 8x NVIDIA A100-SXM4-80GB GPUs	2 × AMD EPYC 7742	64, 128	384	Lustre distributed file system	Rocky Linux 8.10
hpc-pawsey	Pawsey CPU server node	2 × AMD EPYC 7763	128, 256	256	Lustre distributed file system	SUSE Linux Enterprise Server 15 SP5
cloud-S3	Amazon AWS c5a.16xlarge	1 × AMD EPYC 7R32	32, 64 (virtual machine)	128	s3 bucket (standard tier) mounted using s3fs	Ubuntu 24.04
cloud-EBS	Amazon AWS c5a.16xlarge	1 × AMD EPYC 7R32	32, 64 (virtual machine)	128	EBS SSD (gp3, 2,000 GB, IOPS 3000, 125 MB/s)	Ubuntu 24.04
cloud-FsX	Amazon AWS c5a.16xlarge	1 × AMD EPYC 7R32	32, 64 (virtual machine)	128	fsx for Lustre (persistent SSD, 125 MB/s/TiB, 12 TB, no compression, 1,500 MB/s)	Ubuntu 22.04
server-hdd	Server with HDD	2 × Intel Xeon Gold 6154	36, 72	384	12 × 10 TB HDD drives (RAID6, ext4)	Ubuntu 22.04
server-nfs	Server with an NFS mount	2 × Intel Xeon Silver 4114	20, 40	384	NFS mount of a Synology NAS (12 HDD, RAID10, ext4)	Ubuntu 18.04
server-ssd	Server with NVME SSD	2 x AMD EPYC 7313	32, 64	512	2 × 8 TB Micron 7450 NVME SSD (RAID0, ext4)	Ubuntu 22.04
workstation	A workstation with SSD RAID	2 × Intel Xeon Platinum 8180	56, 112	384	8 × 8 TB Micron 5300 SSD (RAID0, ext4)	Ubuntu 20.04
desktop-nvme-ssd	A gaming desktop with NVME SSD	1 × AMD Ryzen Threadripper 3970X	32, 64	128	3 × 2 TB Samsung SSD 970 EVO Plus NVME SSD drives (RAID0, ext4)	Ubuntu 20.04
desktop-sata-ssd	A desktop with SSD	1 × AMD Ryzen Threadripper 3970X	32, 64	128	Samsung 870 QVO 8 TB SSD (ext4)	Ubuntu 20.04
desktop2-ssd	A desktop with SSD	1 × 11th Gen Intel Core i7-11700F	8, 16	64	Samsung 870 QVO 4 TB SSD (ext4)	Pop!_OS 20.04
desktop3-ssd	A desktop with SSD	1 × 12th Gen Intel Core i9-12900K	16, 24	64	Kingston SNV2S 2 TB NVMe SSD (NTFS)	Ubuntu 22.04 through WSL2 on Windows 11
laptop-nvme-ssd	A laptop with NVME SSD	1 × 13th Gen Intel Core i9-13900H	14, 20	64	Samsung 990 Pros 2 TB NVMe SSD (NTFS)	Ubuntu 22.04
laptop-usb-ssd	A laptop with an external hard drive	1 × 13th Gen Intel Core i9-13900H	14, 20	64	Samsung T7 2 TB external USB-c SSD (exFAT)	Ubuntu 22.04
macmini	Apple MacMini external hard drive	1 × Apple ARM M1	8,8	8	Samsung T7 2 TB external USB-c SSD (exFAT)	macOS 15.1
embedded-system	NVIDIA Jetson Xavier	1 × ARM v8	8, 8	16	Samsung 970 EVO Plus 2 TB NVMe SSD (ext4)	Ubuntu 20.04
phone	Motorola Moto G54 5G	1 × MediaTek Dimensity 7020	8,8	8	Samsung Evo Plus 1 TB microSD (exFAT)	Android 13

### File sizes

The series of sequential current signal values making up the body of each read accounts for the majority of the file size in both POD5 and BLOW5 format. Files that store identical signal data are expected to be similar in size. Comparison of identical POD5/BLOW5 files with matched signal compression methods confirmed that any differences in size are negligible (~0.1% difference; Supplementary Table S1). BLOW5 supports a recently developed lossless compression strategy *ex-zd* [15], which is not currently supported in POD5. *Ex-zd* compression delivered a modest 2.4% saving in BLOW5 file size compared to the best available POD5 compression (Supplementary Table S1). As reported elsewhere, BLOW5 file sizes may be significantly reduced (~40%) via *ex-zd* lossy compression with no impact on analysis performance [15], but this was not considered here, as it is not a like-for-like comparison.

### File reading: sequential access

Sequential access is the simplest and typically the most efficient disk access pattern for reading a file. When reading an ONT signal data file, sequential access means reads are accessed by the disk in the order that they occur in the file (as opposed to random access; see below). This pattern is used during basecalling and DNA/RNA modification calling with ONT’s Dorado software and most other analysis tools where signal reads are handled independently. POD5 uses a technique called “mmap” [16, 17] during sequential disk access, whereas SLOW5/BLOW5 uses traditional input/output (I/O) (Table 1).

**Figure 2:**
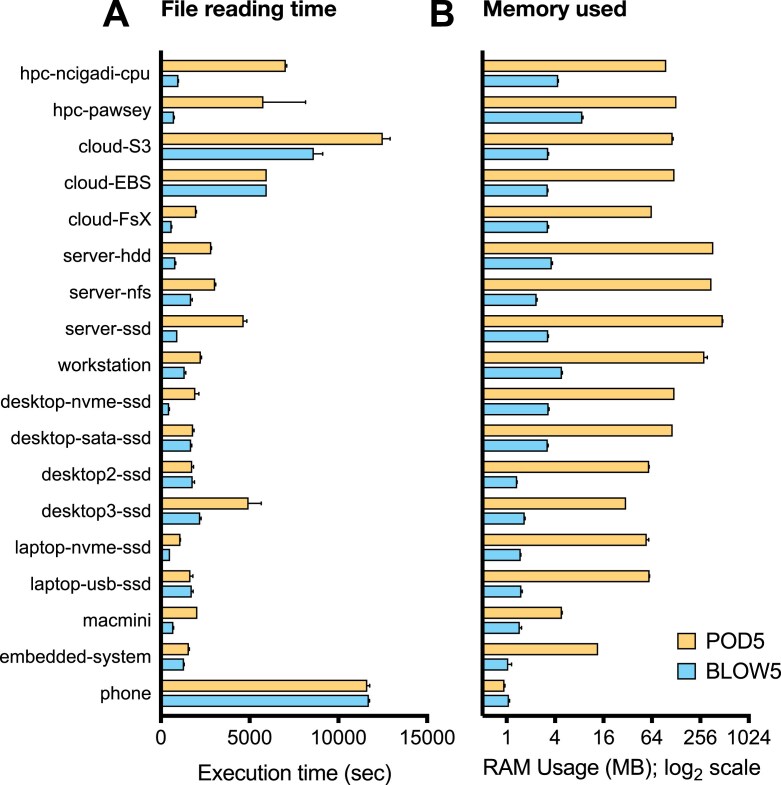
Sequential data access on POD5 versus BLOW5 files. Bar chart shows the time taken (A) and memory used (B) during file reading in a sequential access pattern for an identical dataset (hg2_prom_20x; see Supplementary Table S1) represented in either POD5 (yellow) or BLOW5 (blue) format. The analysis was performed on 18 different computer architectures (see Table 2). Each bar shows the mean of 5 repeated measurements on each system, and error bar shows the range.

To evaluate sequential access, we measured the time and memory required to sequentially read a dataset of ~16 million signal reads in either POD5 or BLOW5 format (hg2_prom_20x; Supplementary Table S1; see Methods). The rate of access varied widely depending on the file type and system architecture. However, across all 18 systems, file reading was either equivalent or faster with BLOW5 (Fig. 2A). Equivalent time was required to read a BLOW5 versus POD5 file on some desktop and laptop computers and an Android smartphone, whereas BLOW5 file reading was up to 3.2× faster on commercial cloud, up to 7.8× faster on high-performance computing (HPC) systems, and 2.9× faster on an Apple desktop computer (Fig. 2A). In addition, the memory used during file reading was dramatically higher for POD5 files on all systems, with the exception of the smartphone (Fig. 2B). The smartphone is an exception here, because it lacks the capacity for mmap and therefore reverts to traditional I/O, which is slower in this context but consumes less memory. Finally, we repeated the experiments across all systems, this time enforcing POD5 reading via traditional I/O rather than mmap. Reading speeds and memory usage were improved on several systems and declined on others, but there was no case in which file reading was significantly faster than for BLOW5 (Supplementary Fig. S1).

Given the large difference in sequential access rates observed on HPC and cloud environments, which are commonly used by the genomics community, we next examined how this may influence performance during ONT basecalling. To emulate a typical scenario, we analyzed human genome sequencing data from a single ONT PromethION flow cell on an academic HPC system (*hpc-ncigadi-gpu*; Table 2; see Methods). We ran ONT’s Dorado software on the native POD5 dataset and a SLOW5-enabled version called slow5-dorado on the equivalent BLOW5 file, using the same basecalling model (dna_r10.4.1_e8.2_400bps_hac@v4.2.0), number of threads for file access (8), and file access batch size (1,000). We observed a ~4.7× difference, with BLOW5 basecalling complete within 32 minutes, compared to 2 hours 24 minutes for POD5 (Table 3). Memory usage during basecalling was also ~12.5× higher for the POD5 (374 GB) versus BLOW5 (30 GB) dataset (Table 3). Given all other variables were matched, this demonstrates how the more efficient sequential data access of the BLOW5 format may manifest in faster basecalling on HPC, cloud, and other similar architectures.

**Table 3:** Comparison of simplex and duplex basecalling performance on an HPC system

Disk access pattern	SLOW5 runtime (hours)	POD5 runtime (hours)	SLOW5 RAM (GB)	POD5 RAM (GB)
Simplex (sequential access)	0.53 (SD 0.01)	2.40 (SD 0.11)	29.9 (SD 0.46)	374.0 (SD 0.60)
Duplex (random access)	6.65 (SD 0.17)	16.69 (SD 2.20)	97.85 (SD 7.94)	118.92 (SD 8.10)

### File reading: random access

The alternative disk access pattern is random access, where signal reads are accessed nonsequentially from within a file, typically by querying specific reads based on their unique identifiers. While this is more complex than sequential access, it is necessary for any analysis where reads are not handled independently or must be accessed in a specific order. For SLOW5/BLOW5, efficient random access is facilitated by an accompanying index file, which specifies the position of each read within the file [11]. In contrast, POD5 uses a 2-step “walker” process, in which the complete file is first traversed to generate a look-up table identifying the location of all batches and rows for each read ID, and then specified reads are fetched from the file using this information (Table 1).

**Figure 3:**
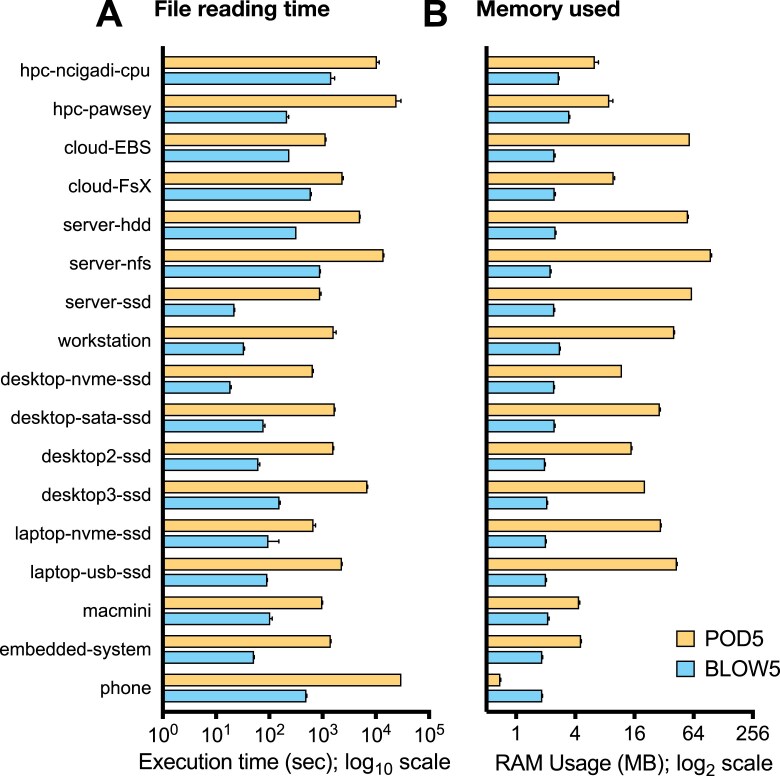
Random data access on POD5 versus BLOW5 files. Bar chart shows the time taken (A) and memory used (B) during file reading in a random access pattern for an identical dataset (hg2_prom_20x; see Supplementary Table S1) represented in either POD5 (yellow) or SLOW5 (blue) format. The analysis was performed on 17 different computer architectures (see Table 2); note the *cloud-S3* system could not be included in the random access benchmark because the POD5 test was so slow that we would be bankrupt by the end of the experiment (not to mention older). Each bar shows the mean of 5 repeated measurements on each system, and error bar shows the range. The x-axis is presented on a logarithmic scale due to the substantial variation in time taken between SLOW5 and POD5.

We measured the time and memory required to read the same POD5/BLOW5 dataset as above, this time using a random access pattern (see Methods). The time taken for file reading again varied between system architectures, but BLOW5 always showed far superior performance (Fig. 3A). The difference in reading time for BLOW5 versus POD5 ranged from 3.9× on the *cloud-FsX* system to 113× on the *hpc-pawsey* system (Fig. 3A). As with sequential access, the memory used during file reading was higher for POD5 files compared to BLOW5 on all systems, with the exception of the smartphone (Fig. 3B). Enforcing the use of traditional I/O for POD5 file-reading reduced the amount of memory required but led to slower performance on all systems (Supplementary Fig. S2).

Given the large differences observed, we next considered an analysis scenario where random access may impact the overall analysis performance—namely, ONT’s duplex basecalling method. During duplex basecalling, independent reads from the complementary strand of a given DNA fragment are analyzed together. Because they are often not adjacent within the input file, a random access pattern is preferable for their retrieval. ONT’s Dorado software retrieves complementary signal reads from a POD5 file using its walker strategy, whereas Slow5-dorado uses a file index to find and retrieve complementary reads. We used both tools to run duplex basecalling on matched POD5/BLOW5 files from a single PromethION flow cell, executed on HPC (*hpc-ncigadi-gpu*; see Methods). Duplex basecalling with POD5/Dorado took 16.7 hours, compared to 6.7 hours (2.5× improvement) with SLOW5/Slow5-dorado, both running with 8 threads for file access (Table 3). Memory usage was again higher for the POD5 (119 GB) versus BLOW5 (98 GB) dataset (Table 3). This analysis demonstrates how more efficient file reading for BLOW5 may manifest in large performance improvements for analysis software using random access patterns, such as duplex basecalling.

### SLOW5 writing performance

A potential advantage of the POD5 format lies in its suitability for parallelized data acquisition. An ONT PromethION P48 (ONT’s largest instrument) has 144,000 data channels, meaning it is theoretically possible for 144,000 DNA/RNA molecules to undergo sequencing simultaneously. At even a fraction of this throughput, it is not feasible to store all reads in memory as they are acquired. This is navigated by writing each read to a POD5 output file in sequential chunks, with chunks from multiple reads acquired in parallel being interspersed within the file (Fig. 1).

In contrast, SLOW5/BLOW5 stores all signal data points for a given read contiguously, meaning a new read cannot start being written to the final output file until the previous read is complete. Parallel data acquisition may instead be handled via a 2-pass strategy in which reads exceeding a certain size are written in sequential chunks to intermediate files, with 1 file per read created temporarily. As sequencing is completed for a given read, its intermediate file is read into memory, appended to the combined BLOW5 output file, and then the intermediate file is removed. Meanwhile, shorter reads are written directly to BLOW5 output files (see Methods).

To evaluate this strategy, we implemented a data simulator, called slowION [18], which mimics data acquisition and reading back (as necessary during live basecalling) from a theoretical nanopore device attached to a given computer (see Methods). We used slowION to simulate ONT data acquisition and reading back at increasing scale in order to find the load at which BLOW5 file writing could no longer keep up. We observed the 2-pass file writing strategy just described to be highly efficient. Our *workstation* computer, which has comparable specifications to an ONT PromethION, could accommodate up to 189,000 data channels before reaching memory limits and our *desktop-nvme-ssd* computer could accommodate 195,000 channels (Table 4). Both computers would therefore be sufficient to accommodate the maximum theoretical data acquisition on a PromethION P48 instrument. Machines with more modest specifications could accommodate fewer channels but were still suitable for relatively high-throughput sequencing. For example, our *laptop-nvme-ssd* computer could accommodate 117,000 parallel channels, sufficient for a PromethION P24 instrument, and our *embedded-system* computer (an NVIDIA Jetson Xavier AGX) could write data for 27,000 channels, theoretically accommodating up to 9× PromethION flow cells on this miniature, low-cost chip (Table 4).

**Table 4:** Evaluating BLOW5 file writing performance on various computer architectures

System name	Max sequencing positions	Max channels	File size per hour (GiB)	RAM (GiB)	Reads (millions)	Samples (billions)
server-hdd	39	117,000	1,434	55.7	7.7	958.0
server-nfs	25	75,000	919	35.8	4.9	614.1
server-ssd	85	255,000	3,125	121.0	16.8	2,087.9
workstation	63	189,000	2,316	89.8	12.4	1,547.5
desktop-nvme-ssd	65	195,000	2,389	92.8	12.8	1,596.6
desktop-sata-ssd	12	36,000	441	17.5	2.4	294.8
desktop2-ssd	11	33,000	404	16.1	2.2	270.2
desktop3-ssd	10	30,000	368	14.6	2.0	245.6
laptop-nvme-ssd	39	117,000	1,433	55.7	7.7	958.0
laptop-usb-ssd	10	30,000	368	14.6	2.0	245.6
macmini	6	18,000	221	2.3	1.2	147.4
embedded-system	9	27,000	331	10.9	1.8	221.1

It is not possible for us to directly compare these results to file-writing performance for POD5, because file writing is handled by ONT’s proprietary MinKNOW software. However, this experiment demonstrates that BLOW5 writing performance would not pose a bottleneck at any sequencing scale that is currently feasible.

### Usability

A file format’s ease of use is an important consideration. Although “usability” is context-dependent and inherently subjective, it is possible to assess relevant aspects like the complexity, compatibility, and stability of a file format and its accompanying software libraries in a semi-quantitative manner. We attempt to do so here.

**Figure 4:**
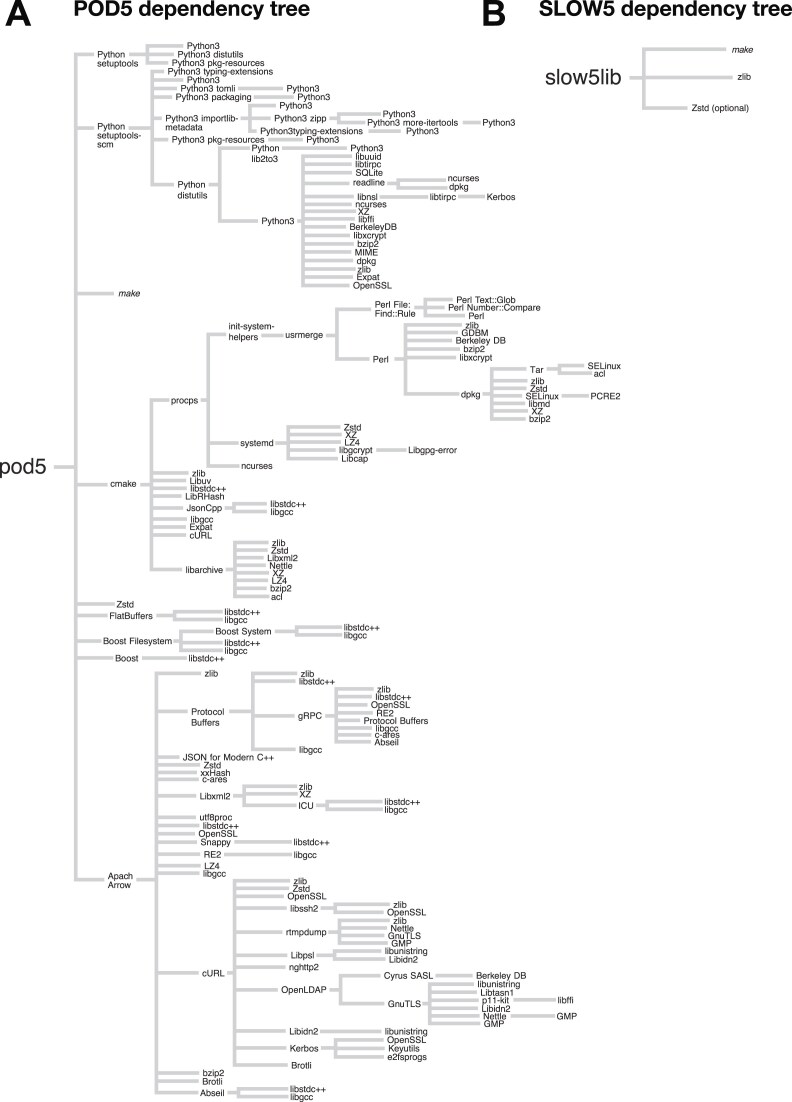
Comparison of POD5 versus SLOW5/BLOW5 software dependencies. The figure depicts the tree of dependencies associated with the POD5 (pod5) and SLOW5 (slow5lib) software libraries. Each dependency subtree is drawn only once; beginning from the trunk, if a subtree is drawn once, it is not drawn again. However, the base node of such eliminated subtree is always included in the tree to indicate its presence and linkage, which means the tree contains some redundant packages. Some dependency names have been simplified for presentation (e.g., zlib1g-dev is simply zlib and libzstd-dev is just zstd). Standard libc dependency (libc6, not to be confused with libgcc) and the C (e.g., gcc) or C++ (e.g., g++) compilers are not included in the tree. Note that Python dependencies are included for POD5 because these are necessary to generate the version of POD5 for *cmake* when compiling the POD5 C++ library. The number of unique dependencies identified at each level in the tree is enumerated in Table 5; when counting dependencies, redundant packages are counted only once.

The number of dependencies for the primary software libraries for reading/writing SLOW5 (slow5lib) and POD5 (pod5) is an important parameter impacting the ease of installation/compilation and compatibility with different user environments. Slow5lib has only 1 mandatory external dependency, zlib, which is natively available on any system (Fig. 4). A second dependency, zstd, enables superior signal compression but is optional only. Both zstd and zlib are self-contained, meaning they introduce no secondary dependencies and can be compiled on any system with common build tools, such as a C compiler and *make*. In comparison, the pod5 library has 9 direct dependencies, including the libraries Apache Arrow, boost, and flatbuffers (Fig. 4). These libraries, in particular Apache Arrow, have their own dependencies, giving rise to a large tree of secondary dependencies, which is summarized in Fig. 4. Following this to a branch depth of 8, we identified 89 unique dependencies for pod5, compared to just 3 for slow5lib (Table 5).

**Table 5:** Summary of dependency tree for POD5 and SLOW5 libraries. The table enumerates the number of unique software dependencies identified at each level of the dependency trees presented in Fig. 4. Each dependency was counted only once; that is, if a given software has already been seen at a higher level in the tree, it is not counted again.

Dependency tree branch depth	POD5 unique dependencies	BLOW5 unique dependencies
1	9	3
2	40	3
3	57	3
4	77	3
5	82	3
6	85	3
7	88	3
8	89	3

This array of dependencies complicates the process of compiling the pod5 library, requiring *cmake*, C++ 17 compiler, conan, ninja-build, and so on. To compare the compilation process between slow5lib and pod5, we measured the time taken to download and install all dependencies on a fresh Docker image using apt (see Methods). For slow5lib, this took 26 seconds and produced a static library size of 578 KiB, whereas the POD5 API required 3 minutes and 27 seconds (8.0× longer), with a static library size of 45 MiB (77× larger; Table 6). In reality, the compilation process will be influenced by the specifics of the user’s environment. However, this experiment provides a simple measure of the complexity involved, with additional complexity equating to a higher risk of encountering incompatibilities or other barriers.

**Table 6:** Comparison of compilation process for POD5 and SLOW5 libraries. On a fresh Docker image of debian:bookworm-slim, the slow5 dependencies were installed from apt, and the slow5 library bench branch was compiled with zstd support. The dependencies for pod5 were downloaded and installed from apt, and *cmake*, followed by *make*, was executed.

Format	Compilation time	Shared library size (.so)	Static library size (.a)
SLOW5	26 seconds	0.56 MiB	1.01 MiB
POD5	207 seconds	16.93 MiB	45.16 MiB

More specialist software developers, as well as maintainers of the file format itself, may interact directly with the library source code. Here, code complexity is important, with simpler code structure being preferable. We applied a range of standard metrics to evaluate the code bases for slow5lib and the pod5 library, excluding their dependencies (Table 7; see Methods). The pod5 code is larger (4.1× more lines), has higher cyclomatic complexity (CCN; 1.27×), and contains a larger number of parameters (1.78×) and functions (4.3×). The only metric on which slow5lib was more complex was its higher CCN per function (1.3×).

**Table 7:** Source code complexity metrics for the SLOW5 and POD5 code base. LOC (lines of code without comments), CCN (cyclomatic complexity number summed over all functions), Tokens (number of tokens—a token is the smallest unit in a program, e.g., operators, keywords, identifiers, separators), Params (total function parameters), Fns (number of functions), and per function averaged statistics. Only includes the main GitHub repository without any dependencies.

Format	LOC	CCN	Tokens	Params	Fns	Avg.LOC/Fn	Avg.CCN/Fn	Avg.Tokens/Fn
slow5	6,916	1,372	39,184	649	252	10.2	2.1	82.0
pod5	28,144	1,749	59,362	1,153	1,072	10.7	1.6	79.1

**Figure 5:**
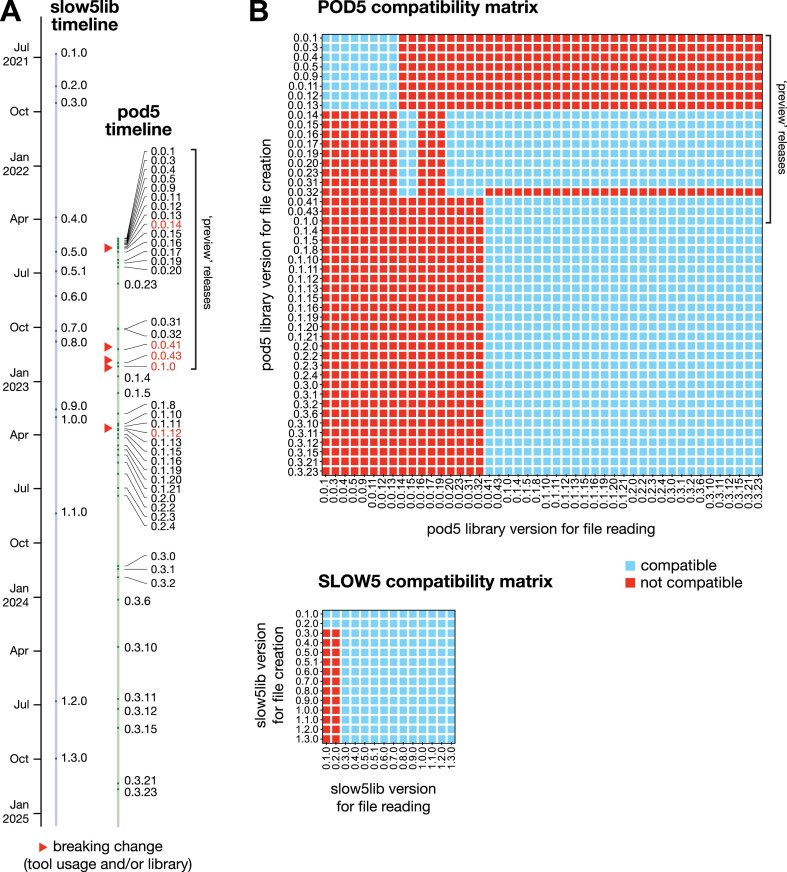
Historical stability of POD5 and SLOW5/BLOW5 formats. (A) The figure depicts a timeline of version releases for the POD5 (pod5) and SLOW5 (slow5lib) software libraries. Red markers indicate releases that introduced a “breaking change,” that is, a change in the library or command line tool usage that requires intervention from the user to restore compatibility with a typical script for file writing and reading (see Methods). (B) The upper figure shows the compatibility of a POD5 file generated with every previous pod5 library version to be read with every other version. Blue squares indicate the file could be read (compatible), and red squares indicate the file could not be read (not compatible). The lower figure presents the equivalent matrix for reading BLOW5 files with all previous slow5lib version releases.

Finally, we assessed the stability or backward/forward compatibility of each code base, which is another important variable for developers. To do so, we generated a POD5 and SLOW5 file using every historic version release of the pod5 library (46 versions) or slow5lib (14 versions; Fig. 5A) and then attempted to read each file with every other library version, creating a file/library compatibility matrix displayed in Fig. 5B (see Methods). For SLOW5, there were no breaks in backward compatibility and a single change in the file format at version 0.3.0, where forward compatibility was broken (Fig. 5B). For POD5, there was a series of breaks in both backward and forward compatibility, meaning that some previous file versions cannot be read with current library versions and vice versa (Fig. 5B). Next, we assessed the code base of each library to identify breaking changes in successive version releases, that is, where usage is disrupted by a change in the library or command line tool, requiring intervention from the user to restore compatibility with basic scripts for file reading and writing (see Methods). While there were no such breaking changes in the history of slow5lib, we identified 5 for pod5, although notably, no breaking changes had been made since April 2023 (version 0.1.12), indicating good stability from this point onward (Fig. 5A).

## Discussion

Here we attempt to determine the optimal format for storing raw signal data from nanopore sequencing experiments. Our comparative evaluation of ONT’s POD5 file format and the open-source alternative, SLOW5, identified several areas where SLOW5 showed clear advantages. File reading was faster for BLOW5 on most computer architectures when using a sequential access pattern and faster on all systems when using a random access pattern; BLOW5 reading used considerably less memory in all scenarios. This manifested in faster, cheaper ONT basecalling with both simplex (sequential access) and duplex (random access) methods. Despite testing on a wide variety of different systems, we could find no instance in which POD5 file reading was significantly faster than BLOW5.

The advantages of BLOW5 for sequential access are the result of its simple row-based layout with contiguous reads, as opposed to the column-oriented and chunk-based layout used in POD5, which necessitates multiple seek operations to retrieve a single read. While the time penalty for performing these seek operations is smaller on systems with fast SSD storage, there is never a scenario where this process could be faster than simply reading sequential rows via traditional I/O, as for BLOW5. Reading a file in a random access pattern is known to be slower than sequential access. However, this is necessary for any analysis where reads are retrieved in a specific order (e.g., based on their genome coordinates). SLOW5 uses a file index to facilitate efficient random access. This is sufficiently fast to enable semi-interactive browsing of signal data aligned to a given genome or transcriptome region using *Squigualiser* [19] and can even be used to extract specified reads from large files on a remote server using *slow5curl* [20]. A comparable indexing strategy is not supported for POD5. This makes it particularly poorly suited for random access, ranging from 3.9× to 113× slower than BLOW5 on the architectures tested here.

While the complex layout used in POD5 creates challenges during file reading, it has supposed advantages for file writing. By storing reads in chunks that may be either adjacent or interspersed in the file, multiple reads can be directly written to the file effectively simultaneously. This parallelization of data acquisition is necessary to support high-volume sequencing on an ONT instrument. However, we show here that this requirement can also be fulfilled via a 2-pass file-writing strategy, which enables highly parallel data acquisition with SLOW5. Our slowION simulation indicates that even a laptop computer can perform BLOW5 file writing at sufficient capacity to accommodate an ONT PromethION P24 instrument at maximum sequencing throughput. SlowION is the simplest implementation of this file-writing strategy, shown here as a proof-of-concept. With further optimization, such as multithreading, we anticipate that the limits of file writing achievable with BLOW5 are likely much higher. An added benefit of the 2-pass approach is that reads are naturally separated by channels and laid onto the final output file in the order in which they were sequenced. This is beneficial when identifying and processing complementary reads during duplex basecalling, which are typically sequenced sequentially on a given channel. Overall, although the chunk-based strategy used in POD5 is a valid solution to the challenge of parallel data acquisition, BLOW5 is similarly suitable to handle the demands of file writing on ONT instruments.

Our semi-quantitative usability analysis identifies some important differences between POD5, SLOW5, and their accompanying libraries. Most notably, the pod5 library has a large tree of direct and secondary dependencies, largely arising from its use of the Apache Arrow software library. In contrast, slow5lib has only 3 direct dependencies—one of which is optional and the other two ubiquitous—and no secondary dependencies. This ensures slow5lib can be easily compiled on any Linux system, whereas pod5 compilation is more likely to encounter conflicts. To alleviate this, ONT provides precompiled pod5 binaries for many common architectures. This is sufficient for most users but cannot cover all possible architectures. Conversely, pod5 binaries are available for Windows systems, whereas slow5lib is not currently supported on Windows. This does not reflect an inherent limitation of the SLOW5/BLOW5 format. Rather, Windows development has not been pursued because Windows Subsystem for Linux (WSL) and other similar solutions are popular among the bioinformatics community, while direct Windows users are relatively rare (if you are Windows user who wants to use SLOW5, please contact us).

In summary, we found SLOW5/BLOW5 to have multiple advantages in terms of performance and usability, and few—if any—parameters on which POD5 was preferable. While ONT instruments do not output data in SLOW5 format directly, users can perform lossless POD5-to-SLOW5 conversion using the software package blue-crab [13]. We continue to maintain SLOW5 and a range of associated software as a community-centric alternative for ONT signal data storage and analysis (see [21]).

## Methods

### File-reading benchmarks

#### Matching conditions

All benchmark experiments were performed on the binary representation of SLOW5, known as BLOW5. This is similar to POD5, which has only a binary version. Performance benchmarks were performed on a large number of different computer systems, over an extended period of time. The pod5 library version 0.3.2 was selected for use in all benchmark experiments, and slow5lib version 1.2.0 was used after patching to match all conditions with the pod5 library (see below). These were the latest library versions available at the time of commencement. ONT developers confirmed that the POD5 file format was stable at that point in time, and therefore no major performance changes were anticipated in the future.

For a fair comparison, it is essential to match all possible conditions, including the compiler versions, compiler optimization flags, and the compression methods. We used precompiled binaries for pod5, provided by ONT, and deduced the compiler versions, compiler optimization flags, and the zstd library version used in those compiled binaries to be gcc/g++ 10, −O3 optimization level, and zstd 1.5, respectively. We therefore compiled slow5lib with those settings to ensure matched conditions. POD5 implemented a newer version of VBZ compression that uses an SIMD-accelerated 2-byte variant of StreamVByte, as opposed to the 4-byte variant of StreamVByte used in slow5lib. We therefore ported this newer VBZ into slow5lib for the purposes of this benchmark. Details of all the commands/conditions that are matched are outlined in Supplementary Note 2.

#### Benchmark programs

The benchmark programs are written in the C/C++ programming language. These benchmark programs perform the following steps repeatedly for a batch of reads at a time.

1. Read the data from the disk, perform decompression, and parse the binary data into memory structures.
2. Perform a simple summation of the signal data values.
3. Output this sum along with metadata.

The time for step 1 was measured by using the “gettimeofday” function called just before and after step 1. Steps 2 and 3 mimic a real program, such as basecalling that consumes loaded data, mainly to prevent compilers from optimizing any unused portions in the code. The peak RAM usage during the lifetime of the benchmark program was measured by using the *maxrss* value from the “getrusage” function. The source of the benchmark program code can be found in the slow5-pod5-bench repository (see [22]).

The code for reading POD5 for sequential access was directly adapted from code used in ONT Dorado for simplex basecalling (DataLoader::load_pod5_reads_from_file in Dorado v0.7). This includes the thread model used in Dorado for accessing POD5 files with multiple threads. The data fields accessed and the order of access to the fields are the same as in Dorado (see Supplementary Note 1). To ensure a fair comparison, we used the same thread model to access the same data fields in the same order in BLOW5, even though this order of access is not the order in which the data are laid out in a BLOW5 file (and therefore not the most efficient way to read a BLOW5 file). Furthermore, when populating data memory structures, POD5 data types are unchanged, whereas BLOW5 data types are converted to match those in POD5, for which the conversion penalty is also included in time measurements for BLOW5. Some examples are as follows:

- sampling_rate, which is a double in BLOW5, is converted to uint16_t to match POD5.
- channel_number, which is a string in BLOW5, is converted to uint16_t to match POD5.
- The fields range and digitization in BLOW5 are both read and divided to compute the single “scale” value in POD5.

The “walker” strategy for performing random access to reads was also directly adapted from code used in ONT Dorado for duplex basecalling (DataLoader::load_pod5_reads_from_file_by_read_ids function in Dorado v0.7). The sanity of our code implementation for POD5 was verified by POD5 developers (for example, see [23]).

#### Benchmarking procedure

The BLOW5 files were first converted to the VBZ compression to match that in POD5 (see above). For random access benchmarks, a list of 500,000 readIDs was randomly created, with the same list being used for both POD5 and BLOW5. Benchmark programs are compiled with g++10 with −O3 optimization (see above).

We performed the benchmarks on various systems (Table 2) and datasets (Supplementary Table S1). Benchmark experiments were executed using all hardware CPU threads available on the system. A read batch size of 1,000 was used in all cases, as this value is hardcoded in POD5 and thus cannot be changed. Each experiment was executed 5 times, and the average execution time and RAM usage were recorded, as described above.

Mitigating the confounding impact of the operating system disk cache is crucial for a file format benchmark. To do so, we used datasets that are larger than the amount of RAM available on the systems (Supplementary Table S1) and, additionally, cleaned the operating system disk caches (pagecache, dentries, and inodes) before every test. In Linux systems where we have root access, this was done by writing the file “/proc/sys/vm/drop_caches.” On Mac, we used the “purge” command. On systems without root access, such as academic supercomputers and the Android smartphone, we wrote and then read large amounts of data (larger than the system RAM available) to fill the disk caches with this mock data.

Example commands are provided in Supplementary Note 2, and benchmarking scripts are available in the slow5-pod5-bench repository (see [22]).

#### Basecaller benchmarks

For basecalling, slow5-dorado v0.3.4 was used for BLOW5 and dorado 0.3.4 for POD5. For simplex basecalling, the basecalling was performed with the high-accuracy model on the dataset hg2_prom_20x. For duplex basecalling, the super-accuracy model was used on the hg2_prom_duplex dataset. All basecalling experiments were performed on the hpc-ncigadi-gpu academic supercomputer with 8 NVIDIA A100 GPUs. As we used prebuilt binaries, the conditions mentioned above are not perfectly matched. However, the conditions favor POD5, as we used the less efficient streamVByte variant, older compiler versions, and less aggressive optimization flags in slow5-dorado binaries. Furthermore, in ONT Dorado (both simplex and duplex basecalling), the number of threads used for POD5 reading is hardcoded to the number of GPUs available on the system (8). Thus, we have forced slow5-dorado also to use 8 threads for BLOW5 access. The BLOW5 batch size is also made 1,000 to match the hardcoded value in POD5. As for the previous benchmarks, we cleaned the disk caches before each experiment, and the average across 5 executions was taken. Commands are provided in Supplementary Note 2.

### SLOW5 writing benchmark

We implemented slowION to simulate the data acquisition process using SLOW5 files, including writing data to disk and subsequently reading it back to mimic live basecalling. slowION is implemented using C on top of the slow5lib library. slowION spawns 3 distinct thread pools, each with a number of threads equal to the number of sequencing positions, implemented using POSIX threads:

1. **Acquisition threads** simulate data acquisition. Read signals shorter than a predefined length threshold (the chunk size) are written directly to a BLOW5 file. Each thread writes to a separate file, so 1 BLOW5 file is created per position. Signal chunks of longer reads (exceeding the chunk size) are written to temporary binary files, with 1 file created per read.
2. **Merge threads** monitor for completed reads in temporary binary files, read them, append them to a separate BLOW5 file, and delete the temporary files. Here, 1 BLOW5 is created for each thread (sequencing position) and is separate from the files used by threads in step 1 above.
3. **Read-back threads** simulate live basecalling by reading completed reads from BLOW5 files. Again, 1 thread is used per sequencing position, which is responsible for the 2 respective BLOW5 files generated from steps 1 and 2 above.

If any thread in any pool fails to complete its task within the real-time constraint, a warning is generated. To ensure that data are truly written to disk (and not just cached in memory), the simulation is run for 1 hour on each system, with total data volume far exceeding available RAM. We repeat the simulation while incrementally increasing the number of sequencing positions in slowION until warnings begin to appear. By gradually increasing the number of simulated positions in parallel, we identify the point at which the first warning appears (at least 1 warning). This is considered the ceiling for parallel data acquisition on a given system. The occurrence of even a single warning is treated as a failure under this strictest evaluation criterion, although in practice, a system can tolerate such incidental warnings. The exact commands used are provided in Supplementary Note 2.

To understand the process, consider the following example:

Using a chunk size of 200,000 signal values, which are 2 bytes each, a PromethION with 144,000 data channels translates to ~54 GiB of memory. This is comfortably accommodated in the RAM of a PromethION compute tower. Given a 5-kHz sampling rate and 400-bps translocation speed, 200,000 signal samples translates to ~16 kbases. In a given sequencing run, a lot of reads are shorter than 16 kbases and therefore fit inside a single chunk. Such reads are first cached in the RAM, then directly written to a BLOW5 file. Only the reads longer than this threshold will go through 2-pass file writing. These longer reads are first written to an intermediate temporary binary file, with 1 temporary file created for each read. As soon as the acquisition of a long read is completed, the temporary file is read into memory and appended to a BLOW5 file, and the temporary file is removed.

### Evaluating usability

#### Dependencies and compilation

To create the software dependency trees for the slow5lib C library and the pod5 C/C++ library, the direct dependencies were first identified from the source code and documentation. For each direct dependency identified, the *apt info* command on Debian 12 (Bookworm) was then executed, and the “Depends” tag of the output was used to identify its dependencies, forming the second layer in the dependency tree. This was repeated for all the dependency levels until we reached a package with no further dependencies. When counting the number of dependencies, we only counted unique software—that is, for a given level, we did not recount a dependency if it had been seen at a previous level of the tree.

The time for building the slow5 C library (with ztsd support) and pod5 C/C++ libraries was measured separately by performing the building steps inside fresh Docker instances. The experiments were performed on the server-nfs system (Table 1) using Debian 12 (Bookworm) as the Docker image. The execution time for the steps (including the installation of dependencies using the apt package manager) was measured using the GNU time utility. Sizes of the generated library files were measured using the *du* command. Further details are provided in Supplementary Note 2.

#### Source code complexity

Source code complexity metrics were measured using lizard version 1.17.17 [24]. For source code complexity measurements, only the source code files inside the GitHub repository required to build the C/C++ library were included. External dependencies, documentation, tests, benchmarks and examples, and Python wrappers were not included. Comments and white space were not included for line counts. Further details are provided in Supplementary Note 2.

#### Compatibility and stability

To evaluate the stability and backward/forward compatibility of POD5 and SLOW5, we developed a simple “test program” that sums up the picoampere converted raw signal for SLOW5 and POD5 separately. We then prepared a BASH script that iteratively tests the compatibility of a given library version with a file created by a different version. The testing process is as follows:

1. Install all software library versions. For SLOW5, we downloaded the slow5lib source from GitHub and compiled it. For POD5, we used precompiled library binaries downloaded from GitHub.
2. Install all toolkit versions. For slow5tools, we downloaded binaries from the slow5tools GitHub repository. For pod5, we installed the pod5 toolkit Python package.
3. Create a POD5/BLOW5 file from each installed version of the relevant toolkit.
4. Compile the “test program” against each library version.
5. Run each “test program” created in step 4 above with all the files created in step 3 above, then check whether the output of the test program is correct.

If the output of the program was correct for a given combination of versions, those versions were deemed to be compatible. If the program failed to run or produced an output that was incorrect, those versions were deemed to be incompatible. Some versions of pod5 perform an internal conversion of one version to another on the disk as a temporary file before reading it; we do not consider this a failure because it is handled internally.

To assess stability, we generated a version release timeline for the SLOW5 and POD5 software libraries and identified “breaking changes.” Our definition of a breaking change is when either the script or test program above had to be modified to work with a given version due to a change in the toolkit command or the C library API. We did not include any cases where the binaries/source code for a particular version was missing in the pod5 repository.

### Blue-crab implementation

A Python tool, blue-crab [13], was developed to allow POD5-to-S/BLOW5 and S/BLOW5-to-POD5 lossless conversion. Using the pod5 and pyslow5 Python libraries, blue-crab can convert many split files of one format to a mirrored folder structure of the other format, or many files to one file. It can be installed with a “pip install blue-crab” command. Extensive testing is carried out to ensure compatibility is maintained with new releases as the POD5 schema changes over time.

## Supplementary Material

giaf118_Supplemental_File

giaf118_Authors_Response_To_Reviewer_Comments_Original_Submission

giaf118_GIGA-D-25-00254_Original_Submission

giaf118_GIGA-D-25-00254_Revision_1

giaf118_Reviewer_1_Report_Original_Submission Wouter De Coster -- 7/28/2025

giaf118_Reviewer_1_Report_Revision_1 Wouter De Coster -- 9/12/2025

giaf118_Reviewer_2_Report_Original_Submission Jan Voges -- 8/12/2025

## Data Availability

The BLOW5 files (zlib+svb-zd compression) of the data are available through the ENA database under the BioProject PRJEB64652 with accessions listed below. These BLOW5 files can be converted to BLOW5 (vbz) and POD5 versions, as explained in Supplementary Note 2. hg2_prom_20x dataset: ERR12997167 hg2_prom_40x dataset: ERR12997168 hg2_prom_duplex dataset: ERR13475640 uhr_rna_prom dataset: ERR12997170

## Additional Files

**Supplementary Table S1**. Summary of datasets used during benchmarking experiments.

**Supplementary Fig. S1**. Sequential data access on POD5 versus BLOW5 files extended. Bar chart shows the time taken (A) and memory used (B) during file reading in a sequential access pattern for an identical dataset (hg2_prom_20x; see Supplementary Table S1) represented in either POD5 or BLOW5 format, as per Fig. 2. For POD5, file reading was measured for the mmap method used by default (yellow) and traditional I/O (orange). The analysis was performed on 18 different computer architectures (see Table 2). Each bar shows the mean of 5 repeated measurements on each system, and error bar shows the range.

**Supplementary Fig. S2**. Random data access on POD5 versus BLOW5 files. Bar chart shows the time taken (**A**) and memory used (**B**) during file reading in a random access pattern for an identical dataset (hg2_prom_20x; see Supplementary Table S1) represented in either POD5 or SLOW5 format, as per Fig. 3. For POD5, file reading was measured for the mmap method used by default (yellow) and traditional I/O (orange). The analysis was performed on 17 different computer architectures (see Table 2); note the *cloud-S3* system could not be included in the random access benchmark because the POD5 test was so slow that we would be bankrupt by the end of the experiment (not to mention older). Each bar shows the mean of 5 repeated measurements on each system, and the error bar shows the range. The x-axis is presented on a logarithmic scale due to the substantial variation in time taken between SLOW5 and POD5.

**Supplementary Note 1**. Field access order.

**Supplementary Note 2**. Detailed methods.

## Abbreviations

HPC: high-performance computing; I/O: input/output; IPC: Interprocess Communication; ONT: Oxford Nanopore Technologies; WSL: Windows Subsystem for Linux.

## Availability of Source Code and Requirements

Project name: slow5-pod5-bench

Project homepage: https://github.com/hasindu2008/slow5-pod5-bench

License: MIT license

SciCrunch RRID:SCR_027155

bio.tools ID: https://bio.tools/slow5tools

Bluecrab: https://github.com/Psy-Fer/blue-crab

slowION: https://github.com/hasindu2008/slowion

System requirements

Operating system: Linux or MacOS

Programming language: C/C++

Package management: distributable binaries

Hardware requirements: x86_64 or ARM64 CPU
