# Supplementary material for: The enduring advantages of the SLOW5 file format for raw nanopore sequencing data
Source: Gigascience. 2025 Oct 16;14:giaf118. doi: 10.1093/gigascience/giaf118 (PMC12530089; doi:10.1093/gigascience/giaf118)
Supplement: giaf118_Supplemental_File [file giaf118_supplemental_file.pdf]

SUPPLEMENTARY INFORMATION

The enduring advantages of the SLOW5 file format for raw nanopore sequencing data

Hasindu Gamaarachchi[1,2]*, Sasha Jenner[2], Hiruna Samarakoon[1,2], James M. Ferguson[2], Ira W. Deveson[2,3]*

1. School of Computer Science and Engineering, University of New South Wales, Sydney, NSW, Australia.

2. Genomics and Inherited Disease Program, Garvan Institute of Medical Research, Sydney, NSW, Australia.

3. St Vincent's Clinical School, Faculty of Medicine, University of New South Wales, Sydney, NSW, Australia.

* Correspondence: hasindu@garvan.org.au, i.deveson@garvan.org.au

Supplementary Table S1. Summary of datasets used during benchmarking experiments. p2
Supplementary Figure S1. Sequential data access on POD5 vs BLOW5 files extended. p3
Supplementary Figure S2. Random data access on POD5 vs BLOW5 files. p4
Supplementary Note 1: Field access order. p5
Supplementary Note 2: Detailed Methods. p6-9

Supplementary Table S1. Summary of datasets used during benchmarking experiments

Dataset name	Description	N reads	Total signal Samples	BLOW5 (VBZ) Size	BLOW index size	POD5 (VBZ) Size	BLOW5 (ex-zd) size
hg2_prom_20x	HG002 PromethION LSK114 at 5kHz	16.1 million	903.2 billion	739 GiB	0.8 GiB	740 GiB	723 GiB
hg2_prom_40x	HG002 PromethION LSK114 at 5kHz	21.7 million	2035.7 billion	1659 GiB	1.1 Gib	1661 GiB	1622 GiB
hg2_prom_duplex	HG002 PromethION LSK114 ‘high duplex’ at 5kHz	4.9 million	895.4 billion	731 GiB	0.2 GiB	733 GiB	716 GiB
uhr_rna_prom	Universal human RNA; direct RNA004	16.4 million	641.1 billion	549 GiB	0.8 GiB	549 GiB	539 GiB

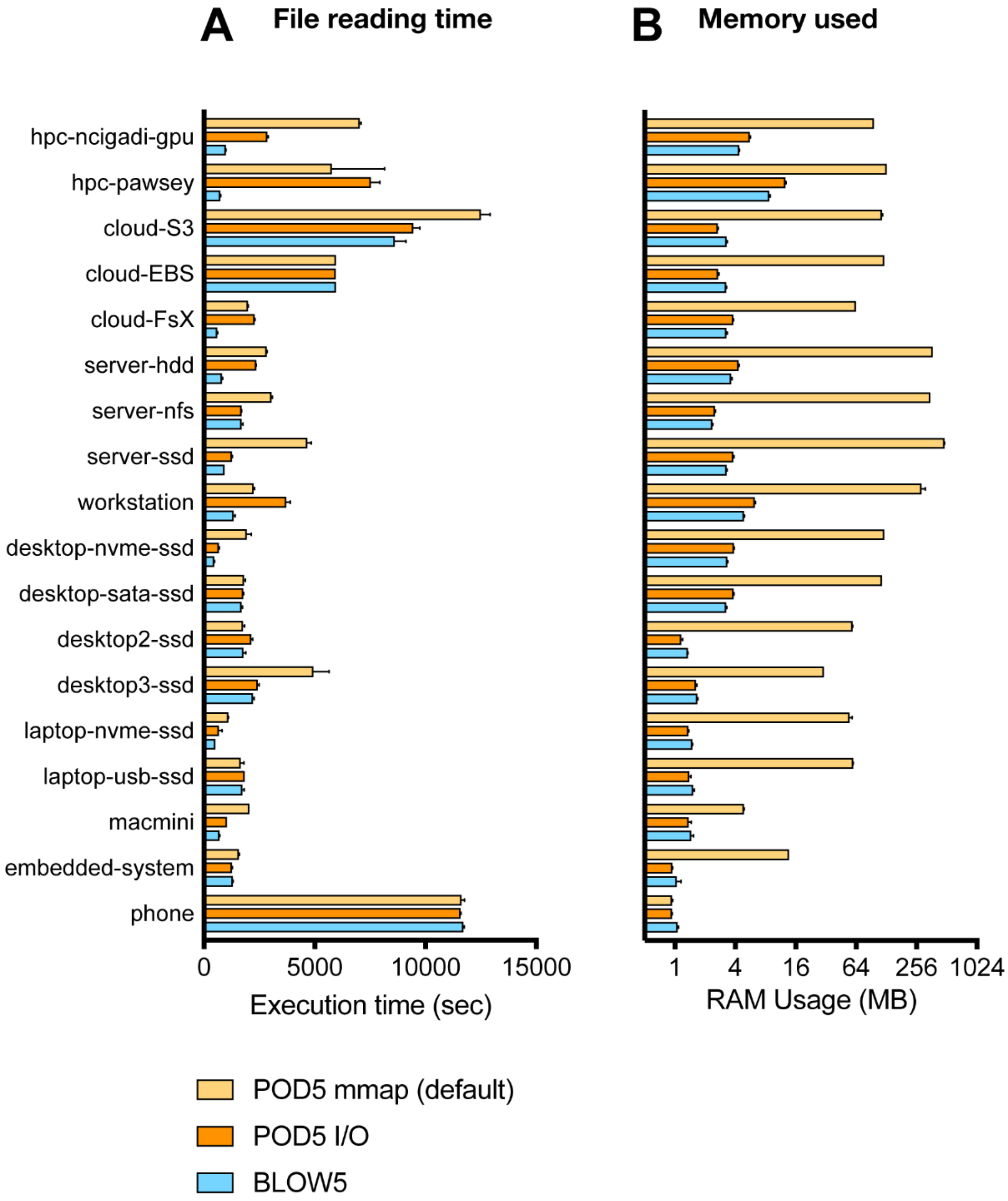

Supplementary Figure S1. Sequential data access on POD5 vs BLOW5 files extended. Bar chart shows the time taken (**A**) and memory used (**B**) during file reading in a sequential access pattern for an identical dataset (hg2_prom_20x; see **Table S1**) represented in either POD5 or BLOW5 format, as per **Figure 2.** For POD5, file reading was measured both for the mmap method used by default (yellow) and traditional I/O (orange). The analysis was performed on 18 different computer architectures (see **Table 2**). Each bar shows the mean of five repeated measurements on each system and error bar shows the range.

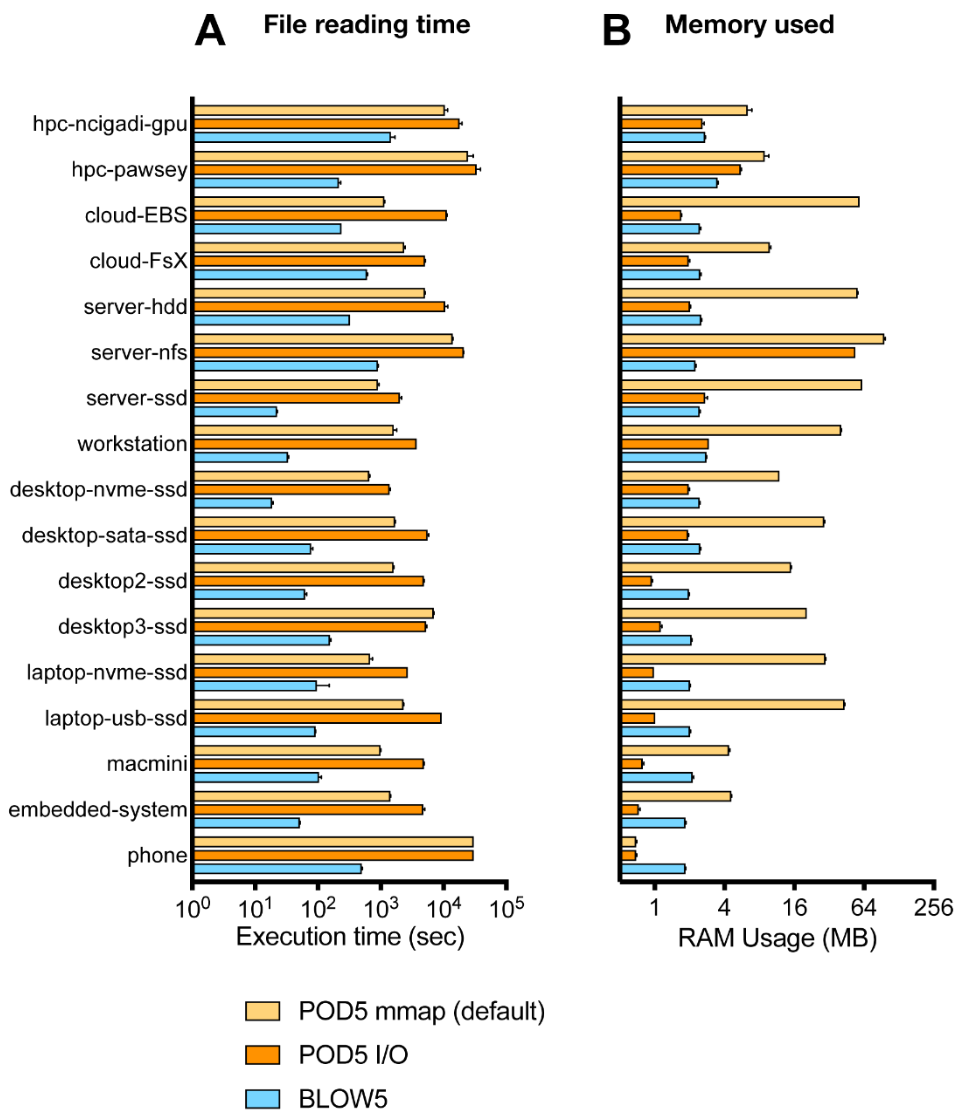

Supplementary Figure S2. Random data access on POD5 vs BLOW5 files. Bar chart shows the time taken (**A**) and memory used (**B**) during file reading in a random access pattern for an identical dataset (hg2_prom_20x; see **Table S1**) represented in either POD5 or SLOW5 format, as per Figure 3. For POD5, file reading was measured both for the mmap method used by default (yellow) and traditional I/O (orange). The analysis was performed on 17 different computer architectures (see **Table 2**); note the *cloud-S3* system could not be included random access benchmark because the POD5 test were so slow we would be bankrupt by the end of the experiment (not to mention older). Each bar shows the mean of five repeated measurements on each system and error bar shows the range. The a-axis is presented on a logarithmic scale due to the substantial variation in time taken between SLOW5 and POD5.

Supplementary Note 1: Field access order

The fields accessed (in order) are as below:

1. run_acquisition_start_time_ms
2. sample_rate
3. read_id
4. num_samples
5. raw_signal
6. start_sample
7. calibration_scale (scaling)
8. calibration_offset (offset)
9. read_number
10. well (mux)
11. channel (channel number)
12. acquisition_id (run_id)
13. flowcell_id
14. sequencer_position (position_id)
15. experiment_name (experiment_id)

This is the order used in Dorado v0.7 (https://github.com/nanoporetech/dorado/blob/9ac85c65fc873a956bda00b2f5608b2bf72d9e7c/dorado/data_loader/DataLoader.cpp#L876)

Supplementary Note 2: Detailed Methods

Format/compression conversion

```
# conversion to the compression type ${REC_MTD}_${SIG_MTD} (e.g., REC_MTD=zstd, SIG_MTD=svb16-zd)
slow5tools view reads.blow5 -c ${REC_MTD} -s ${SIG_MTD} -o reads_${REC_MTD}_${SIG_MTD}.blow5 -t40
slow5tools index reads_${REC_MTD}_${SIG_MTD}.blow5

# converting to BLOW5->POD5
blue-crab s2p reads.blow5 -o reads.pod5 -p 40
```

Versions:

- for zstd+svb16-zd (vbz): slow5tools vbz branch [https://github.com/hasindu2008/slow5tools/tree/vbz commit 8a366bf6dffe0c94fd0ec148cca22f09e47c31e5].
- zstd+svb-zd and zstd+ex-zd: slow5tools 1.3.0
- blue-crab 0.1.0

File size measurements

```
# measure size in bytes
du -b <file>
```

Disk cache cleaning

- For systems with root access: https://github.com/hasindu2008/biorand/blob/master/clean_fscache.c
- For systems without root access: https://github.com/hasindu2008/biorand/blob/master/clean_fscache2.c

Reading

```
# slow5 sequential (run_seq.sh available under slow5 directory in the slow5-pod5-bench repository)
./run_seq.sh zstd-svb16-zd.blow5 <num-cpu-threads> 1000 cxx

# pod5 sequential using default mmap I/O (run_seq.sh available under pod5 directory in the slow5-pod5-bench
repository)
./run_seq.sh reads.pod5 <num-cpu-threads> mmap

# pod5 sequential forced to use traditional I/O (run_seq.sh available under pod5 directory in the slow5-pod5-
bench repository)
./run_seq.sh reads.pod5 <num-cpu-threads> io

# getting 500k random readIDs
slow5tools skim --rid zstd-svb16-zd.blow5 | sort -R | head -500000 > ridlist.txt

# slow5 random (run_rand.sh available under slow5 directory in the slow5-pod5-bench repository)
./run_rand.sh zstd-svb16-zd.blow5 ridlist.txt <num-cpu-threads> 1000 cxx

# pod5 random using default mmap I/O (run_rand.sh available under pod5 directory in the slow5-pod5-bench
repository)
./run_rand.sh reads.pod5 ridlist.txt <num-cpu-threads> 1000 mmap

# pod5 random forced to use traditional I/O (run_rand.sh available under pod5 directory in the slow5-pod5-
bench repository)
./run_rand.sh reads.pod5 ridlist.txt <num-cpu-threads> 1000 io
```

SUPPLEMENTARY INFORMATION

Basecalling

```
# slow5 simplex basecalling
/usr/bin/time -v slow5-dorado basecaller dna_r10.4.1_e8.2_400bps_hac@v4.2.0 simplex_zstd-svb-zd.blow5 --emit-fastq --slow5_threads 8 --slow5_batchsize 1000  -x cuda:all > reads.fastq

# pod5 simplex basecalling
/usr/bin/time -v dorado basecaller dna_r10.4.1_e8.2_400bps_hac@v4.2.0 simples.pod5 --emit-fastq  -x cuda:all > reads.fastq

# slow5 duplex basecalling
/usr/bin/time -v slow5-dorado duplex dna_r10.4.1_e8.2_400bps_sup@v4.2.0 duplex_zstd-svb-zd.blow5 --slow5_threads 8 --slow5_batchsize 1000  -x cuda:all  > reads.bam

# pod5 random
/usr/bin/time -v dorado duplex dna_r10.4.1_e8.2_400bps_sup@v4.2.0 duplex.pod5 -x cuda:all > reads.bam
```

Versions:

- slow5-dorado v0.3.4
- Dorado v0.3.4

Writing

```
# simulate for an hour at a 5000 Hz sampling rate using the specified number of positions with 3000 channels per position
./slowION -p <num-seq-positions> -c 3000 -T 3600 -f 5000
```

Versions:

- slowION v0.1.0

Dependency tree

```
# tree.sh is available under the misc directory in the slow5-pod5-bench repository, which can be used to get the dependencies for a given package.

# getting the dependencies for the example package libflatbuffers-dev
tree.sh libflatbuffers-dev
```

versions:

- slow5lib v1.2.0
- pod5 v0.3.2
- Docker version 26.1.3; *debian:bookworm-slim* image (Debian 12)

Notes:

- When drawing the dependency tree, dependency names have been simplified (e.g., zlib1g-dev is simply zlib and libzstd-dev is just zstd).
- Standard libc dependency (*libc6*, not to be confused with libgcc) is not included in the tree. This is because *libc6* is at the leaf of almost every library. For instance, though zlib and zstd have *libc6* at the end, it is not drawn.

SUPPLEMENTARY INFORMATION

- Each dependency subtree is drawn only once. We begin from the bottom, and if a subtree is drawn once, it is not drawn again. As an example, *libstdc++* under Abseil has *libgcc* dependency listed, but above this, any other libstdc++ nodes do not have this libgcc.
- The C (e.g., gcc) or C++ (e.g., g++) compilers are not included in any of the trees.
- Note that Python dependencies are included for POD5, not because we included the Python wrapper for POD5, but because these are necessary to generate the version of POD5 for *cmake* when compiling the POD5 C++ library.

Source code compilation

```
## slow5
/usr/bin/time -v apt-get -y install zlib1g-dev libzstd-dev make gcc
/usr/bin/time -v make zstd=1
du -b lib/libslow5.so
du -b lib/libslow5.a

## pod5
/usr/bin/time -v apt-get -y install ca-certificates lsb-release wget
/usr/bin/time -v wget "https://apache.jfrog.io/artifactory/arrow/$(lsb_release --id --short | tr 'A-Z' 'a-
z')/apache-arrow-apt-source-latest-$(lsb_release --codename --short).deb"
/usr/bin/time -v apt-get -y install "./apache-arrow-apt-source-latest-$(lsb_release --codename --short).deb"
/usr/bin/time -v apt-get update
/usr/bin/time -v apt-get -y install libarrow-dev libflatbuffers-dev libzstd-dev cmake libboost-dev libboost-
filesystem-dev python3-setuptools-scm python3-setuptools git
/usr/bin/time -v python3 -m setuptools_scm
/usr/bin/time -v python3 -m pod5_make_version
mkdir build && cd build
/usr/bin/time -v cmake ..
/usr/bin/time -v make
du -b c++/libpod5_format.so
du -b c++/libpod5_format.a
```

versions:

- slow5lib bench branch [https://github.com/hasindu2008/slow5lib/tree/bench commit c254055ea7811ce3daec541141a8608d5dc15243]
- pod5 v0.3.10
- Docker version 26.1.3; *debian:bookworm-slim* image (Debian 12)

Notes:

- See inside the *docker_slow5.sh* under the *misc* directory of the *slow5-pod5-bench* repository for detailed steps of slow5 compilation
- See inside the *docker_pod5.sh* under the *misc* directory of the *slow5-pod5-bench* repository for detailed steps of pod5 compilation
- reported times for compilation are the sum of all steps that were measured by the time command above
- pod5 v0.3.10 was used instead of pod5 v0.3.2 (the version used for benchmarks), because we could not get this v0.3.2 compiled
- The reason for using Docker is that pod5 library needs specific compiler versions and many packages that were tedious to install without root access. slow5lib is also built inside a Docker for fair comparison of the building time.

Source code complexity

```
## lizard.sh is available under the misc directory in the slow5-pod5-bench repository

# slow5
lizard.sh Makefile include/ src/ thirdparty/streamvbyte/Makefile thirdparty/streamvbyte/include/
thirdparty/streamvbyte/src/

# pod5
lizard.sh conanfile.py pod5_make_version.py, CMakeLists.txt, cmake/* c++/CMakeLists.txt c++/pod5_format/
third_party/include/
```

versions:

- slow5: https://github.com/hasindu2008/slow5lib/archive/refs/tags/v1.2.0.tar.gz
  pod5: https://github.com/nanoporetech/pod5-file-format/archive/refs/tags/0.3.2.tar.gz
- lizard version 1.17.17 [https://github.com/terryyin/lizard]

Notes:
For slow5, *makefile* source code is included because that is the preferred option. Optional *cmake* is not included for slow5. For pod5, no *make*-based method exist; thus, *cmake* source code is included. For pod5, we also include conanfile.py and pod5_make_version.py, because they are required to build the C++ version. Source code inside third-party directories is included for both slow5 and pod5, because they contain source inbuilt to the repository, which are not considered external dependencies.

Stability/Compatibility

```
## slow5
cd <slow5-pod5-bench_repository>/slow5/compat/
./run.sh

# pod5
cd <slow5-pod5-bench_repository>/pod5/compat/
./run.sh
```
