# Supplementary material for: The enduring advantages of the SLOW5 file format for raw nanopore sequencing data
Source: Gigascience. 2025 Oct 16;14:giaf118. doi: 10.1093/gigascience/giaf118 (PMC12530089; doi:10.1093/gigascience/giaf118)
Supplement: giaf118_Authors_Response_To_Reviewer_Comments_Original_Submission [file giaf118_authors_response_to_reviewer_comments_original_submission.pdf]

Author's Response To Reviewer Comments

Below is a point-by-point response to the reviewer comments. All significant edits have been highlighted in our revised manuscript document.

Reviewer #1

1.1. The authors describe the SLOW5 format and its benefits compared to the standard POD5 format for storing raw sequencing data from nanopore sequencers. The paper is well written and easy to understand. The advantages of the SLOW5 format are clear, and the comparison is adequately executed and described. However, the developers seem unable to persuade others to adopt it widely, and change might need to come from ONT themselves, who may be most concerned about disrupting their existing workflows, especially for parallel writing during sequencing. Nevertheless, the authors seem to have also addressed that issue, as demonstrated with a simulation experiment.

Please find my specific suggestions below.

Sincerely,
Wouter De Coster

We thank Dr De Coster for their constructive assessment of our manuscript. Regarding SLOW5 adoption – we've had many discussions with ONT developers over the years but have been unable to convince them of the merits of a simple, efficient file format. By putting our arguments and clear evidence out there for the community to see, in the form of the current article, we hope this may prompt others to have similar conversations with ONT, and we might eventually persuade them.

1.2. Major:
While I understand that the software name SLOW5 was an initial variation of the FAST5 format, I don't think that the words 'slow' or the number '5' are particularly appropriate descriptions or helpful in making a case for using the file format, as it is neither slow nor related to HDF5. However, once a name is chosen, I understand the reluctance to change it. Additionally, it seems the evaluations are conducted using the binary BLOW5 format. Wouldn't it then make more sense to emphasize BLOW5 in the text and title?

'SLOW5' was intended as a memorable, tongue-in-cheek, name, rather than any technical description of the format. In our opinion, a catchy name like this acts as a useful meme, and is beneficial for attention and adoption. In addition, changing the name now would cause compatibility issues with user's software, pipelines, etc, and is therefore inconsistent with our commitment to stability and simplicity.

The reviewer is correct that we used binary SLOW5 (BLOW5) during benchmarking, not the ASCII version. We use 'SLOW5' as an umbrella term for both the ASCII and binary versions. However, we appreciate that this could cause some confusion. In our revised manuscript we have clarified the wording throughout the manuscript, figures and legends, to make it clear when we are referring to SLOW5 vs BLOW5 files. We prefer to keep 'SLOW5' in the title.

Actions: language clarified throughout manuscript and figures regarding usage of BLOW5 vs SLOW5 file versions.

1.3. Minor:
I would italicize the 'make' tool for users unfamiliar with build tools in the Usability section, as it is a rather strange sentence if reading 'make' as a verb, not a tool. Perhaps the same could be applied to other dependencies in that section for consistency. Then again, the primary target audience will probably understand what 'make' means in this context.

Good suggestion; we have made this change.

Actions: ‘make’ tool italicised throughout manuscript and figures.

1.4. There is a typo in the benchmarking procedure section: 'confoudning'.

Thank you for picking this up.

Actions: typo corrected.

Reviewer #2:

2.1. The manuscript builds on the authors' previous work introducing the SLOW5 format for Oxford Nanopore signal data as an improvement over the FAST5 format. Since then, Oxford Nanopore Technologies (ONT) has introduced its own new format, POD5. This paper directly compares SLOW5 and POD5.

The authors claim that SLOW5 provides higher reading speeds for both sequential and random access, writing speeds sufficient to keep pace with data acquisition in sequencing machines, comparable file sizes with no significant storage penalty, a simpler implementation with fewer dependencies.

The paper is clearly written, includes extensive supplementary information, and references the source code for all tools used in the experiments.

We thank the reviewer for their constructive assessment of our manuscript.

2.2. Sequential access performance: To me it is unclear whether SLOW5's advantage in sequential access originates from its file layout or from the use of mmap I/O versus traditional I/O. A small ablation study, forcing both SLOW5 and POD5 tools to use the same I/O method on platforms with currently large performance differences, would clarify where the performance gain originates from.

At the reviewer’s suggestion, we have repeated both file-reading benchmark experiments but this time forcing POD5 to use traditional I/O rather than mmap to read the file. Performance is variable between different systems, but we find no situation where POD5 file reading is significantly faster than BLOW5 file reading, regardless of the reading method (I/O vs mmap), access pattern (sequential vs random) or machine used. Therefore, POD5’s sub-optimal performance reflects inherent properties of the file format, rather than a difference in the file-reading strategy.

Actions: new file-reading benchmarks with POD5 forced to use I/O are included in Supplementary Figures 1 & 2 and reported in the main RESULTS section.

2.3. Figure 4: While POD5's dependency structure is indeed more complex than that of slow5lib, the current tree representation exaggerates this complexity. Many common packages (e.g., Python, zlib) appear multiple times as dependency of multiple other packages. A dependency graph where each package appears only once would be a more informative representation.

The reviewer is correct that the dependency tree inevitably contains some redundancies. However, when counting dependencies, we are careful to only count unique dependencies – i.e. we do not count the same package twice. The number of unique dependencies at each layer of the tree is enumerated in Table 5, which is equivalent to the dependency graph suggested by the reviewer. All mentions of dependency numbers within the main text refer to unique dependencies.

Actions: Table 5 reports the number of unique dependencies at each level of the dependency tree, equivalent to the suggested dependency graph. The legend for Figure 4 clarifies the handling of redundant packages.

2.4. Figure 5: POD5 versions prior to 0.1.0 appear to be preview releases (and are even marked as such on GitHub). Breaking changes during early previews are normal, so including them in the same visual space as stable versions risks being misleading.

To clarify this point, we have included additional labels on Figure 5 to distinguish between ‘preview’ release and stable versions of POD5. We note that POD5 had been launched at ONT’s London Calling conference and users were being actively encouraged to trial the format during this ‘preview’ stage, which is why we have included it in our evaluation.

Actions: labels added to Figure 5 to distinguish stable vs pre-release POD5 versions.

2.5. Figure 5: Breaking change at version 0.1.12: The timeline indicates a breaking change at POD5 version 0.1.12 which seems particularly relevant as the latest breaking change after version 0.1.0. However, this change is not reflected in the POD5 compatibility matrix on the right. An explanation of what type of breaking change occurred would clarify its impact and help readers assess compatibility risk.

The breaking change at version 0.1.12 was a change to the POD5 API, in which its usage was modified. Such a change requires intervention to fix any tools or pipelines that were relying on the POD5 API, given the modified usage, which we classify as a ‘breaking change’. This is different to the compatibility matrix which shows the capacity of a file written by a past / future version of the API to be read by a current version. These analyses are related but not equivalent – we have sought to clarify the difference within the Figure 5 legend and METHODS section.

Actions: extra detail added to METHODS section and legend of Figure 5 to clarify the difference between the breaking change vs compatibility matrix analyses.

2.6. Random access "walker strategy": A brief explanation comparing it to SLOW5's index-file approach would improve accessibility without requiring readers to consult external documentation.

At the reviewer’s suggestion, we have included a brief explanation of the ‘walker’ strategy used by POD5 for random access file reading within the RESULTS section.

Actions: information on ‘walker’ strategy included in the revised manuscript.

Cookie Preference Center

We use cookies which are necessary to make our site work. We may also use additional cookies to analyze, improve and personalize our content and your digital experience. For more information, see our Cookie Policy.

You may choose not to allow some types of cookies. However, blocking some types may impact your experience of our site and the services we are able to offer. See the different category headings below to find out more or change your settings.

Allow all

Manage Consent Preferences

Strictly Necessary Cookies

Always active

These cookies are necessary for the website to function and cannot be switched off in our systems. They are usually only set in response to actions made by you which amount to a request for services, such as setting your privacy preferences, logging in or filling in forms. You can set your browser to block or alert you about these cookies, but some parts of the site will not then work.

Functional Cookies

These cookies enable the website to provide enhanced functionality and personalisation. They may be set by us or by third party providers whose services we have added to our pages. If you do not allow these cookies then some or all of these services may not function properly.

Performance Cookies

These cookies allow us to count visits and traffic sources so we can measure and improve the performance of our site. They help us to know which pages are the most and least popular and see how visitors move around the site.

Targeting Cookies

These cookies may be set through our site by our advertising partners. They may be used by those companies to build a profile of your interests and show you relevant adverts on other sites. If you do not allow these cookies, you will experience less targeted advertising.

Cookie List

Clear

Apply Cancel
Consent Leg.Interest

Confirm my choices

Powered by onetrust
