# Supplementary material for: The enduring advantages of the SLOW5 file format for raw nanopore sequencing data
Source: Gigascience. 2025 Oct 16;14:giaf118. doi: 10.1093/gigascience/giaf118 (PMC12530089; doi:10.1093/gigascience/giaf118)
Supplement: giaf118_Reviewer_1_Report_Original_Submission — Wouter De Coster -- 7/28/2025 [file giaf118_reviewer_1_report_original_submission.pdf]

**Reviewer Report**

**Title: The enduring advantages of the SLOW5 file format for raw nanopore sequencing data**

**Version: Original Submission** **Date:** 7/28/2025

**Reviewer name: Wouter De Coster**

**Reviewer Comments to Author:**

The authors describe the SLOW5 format and its benefits compared to the standard POD5 format for storing raw sequencing data from nanopore sequencers. The paper is well written and easy to understand. The advantages of the SLOW5 format are clear, and the comparison is adequately executed and described. However, the developers seem unable to persuade others to adopt it widely, and change might need to come from ONT themselves, who may be most concerned about disrupting their existing workflows, especially for parallel writing during sequencing. Nevertheless, the authors seem to have also addressed that issue, as demonstrated with a simulation experiment.Please find my specific suggestions below.Sincerely,Wouter De CosterMajor:While I understand that the software name SLOW5 was an initial variation of the FAST5 format, I don't think that the words 'slow' or the number '5' are particularly appropriate descriptions or helpful in making a case for using the file format, as it is neither slow nor related to HDF5. However, once a name is chosen, I understand the reluctance to change it. Additionally, it seems the evaluations are conducted using the binary BLOW5 format. Wouldn't it then make more sense to emphasize BLOW5 in the text and title?Minor:I would italicize the 'make' tool for users unfamiliar with build tools in the Usability section, as it is a rather strange sentence if reading 'make' as a verb, not a tool. Perhaps the same could be applied to other dependencies in that section for consistency. Then again, the primary target audience will probably understand what 'make' means in this context.There is a typo in the benchmarking procedure section: 'confoudning'.

**Level of Interest**

Please indicate how interesting you found the manuscript: Choose an item.

**Quality of Written English**

Please indicate the quality of language in the manuscript: Choose an item.

**Declaration of Competing Interests**

Please complete a declaration of competing interests, considering the following questions:

- Have you in the past five years received reimbursements, fees, funding, or salary from an organisation that may in any way gain or lose financially from the publication of this manuscript, either now or in the future?

- Do you hold any stocks or shares in an organisation that may in any way gain or lose financially from the publication of this manuscript, either now or in the future?
- Do you hold or are you currently applying for any patents relating to the content of the manuscript?
- Have you received reimbursements, fees, funding, or salary from an organization that holds or has applied for patents relating to the content of the manuscript?
- Do you have any other financial competing interests?
- Do you have any non-financial competing interests in relation to this paper?

If you can answer no to all of the above, write 'I declare that I have no competing interests' below. If your reply is yes to any, please give details below.

I have received travel and accommodation reimbursement from ONT to present at London Calling

I agree to the open peer review policy of the journal. I understand that my name will be included on my report to the authors and, if the manuscript is accepted for publication, my named report including any attachments I upload will be posted on the website along with the authors' responses. I agree for my report to be made available under an Open Access Creative Commons CC-BY license (http://creativecommons.org/licenses/by/4.0/). I understand that any comments which I do not wish to be included in my named report can be included as confidential comments to the editors, which will not be published.

Choose an item.

To further support our reviewers, we have joined with Publons, where you can gain additional credit to further highlight your hard work (see: https://publons.com/journal/530/gigascience). On publication of this paper, your review will be automatically added to Publons, you can then choose whether or not to claim your Publons credit. I understand this statement.

Yes Choose an item.
