# Supplementary material for: The enduring advantages of the SLOW5 file format for raw nanopore sequencing data
Source: Gigascience. 2025 Oct 16;14:giaf118. doi: 10.1093/gigascience/giaf118 (PMC12530089; doi:10.1093/gigascience/giaf118)
Supplement: giaf118_Reviewer_2_Report_Original_Submission — Jan Voges -- 8/12/2025 [file giaf118_reviewer_2_report_original_submission.pdf]

Reviewer Report

Title: The enduring advantages of the SLOW5 file format for raw nanopore sequencing data

Version: Original Submission **Date:** 8/12/2025

Reviewer name: Jan Voges

Reviewer Comments to Author:

Synopsis:The manuscript builds on the authors' previous work introducing the SLOW5 format for Oxford Nanopore signal data as an improvement over the FAST5 format. Since then, Oxford Nanopore Technologies (ONT) has introduced its own new format, POD5. This paper directly compares SLOW5 and POD5.The authors claim that SLOW5 provides higher reading speeds for both sequential and random access, writing speeds sufficient to keep pace with data acquisition in sequencing machines, comparable file sizes with no significant storage penalty, a simpler implementation with fewer dependencies.The paper is clearly written, includes extensive supplementary information, and references the source code for all tools used in the experiments.Comments:- Sequential access performance: To me it is unclear whether SLOW5's advantage in sequential access originates from its file layout or from the use of mmap I/O versus traditional I/O. A small ablation study, forcing both SLOW5 and POD5 tools to use the same I/O method on platforms with currently large performance differences, would clarify where the performance gain originates from.- Figure 4: While POD5's dependency structure is indeed more complex than that of slow5lib, the current tree representation exaggerates this complexity. Many common packages (e.g., Python, zlib) appear multiple times as dependency of multiple other packages. A dependency graph where each package appears only once would be a more informative representation.- Figure 5: POD5 versions prior to 0.1.0 appear to be preview releases (and are even marked as such on GitHub). Breaking changes during early previews are normal, so including them in the same visual space as stable versions risks being misleading.- Figure 5: Breaking change at version 0.1.12: The timeline indicates a breaking change at POD5 version 0.1.12 which seems particularly relevant as the latest breaking change after version 0.1.0. However, this change is not reflected in the POD5 compatibility matrix on the right. An explanation of what type of breaking change occurred would clarify its impact and help readers assess compatibility risk.- Random access "walker strategy": A brief explanation comparing it to SLOW5's index-file approach would improve accessibility without requiring readers to consult external documentation.

Level of Interest

Please indicate how interesting you found the manuscript: Choose an item.

Quality of Written English

Please indicate the quality of language in the manuscript: Choose an item.

Declaration of Competing Interests

Please complete a declaration of competing interests, considering the following questions:

- Have you in the past five years received reimbursements, fees, funding, or salary from an organisation that may in any way gain or lose financially from the publication of this manuscript, either now or in the future?
- Do you hold any stocks or shares in an organisation that may in any way gain or lose financially from the publication of this manuscript, either now or in the future?
- Do you hold or are you currently applying for any patents relating to the content of the manuscript?
- Have you received reimbursements, fees, funding, or salary from an organization that holds or has applied for patents relating to the content of the manuscript?
- Do you have any other financial competing interests?
- Do you have any non-financial competing interests in relation to this paper?

If you can answer no to all of the above, write 'I declare that I have no competing interests' below. If your reply is yes to any, please give details below.

I declare that I have no competing interests.

I agree to the open peer review policy of the journal. I understand that my name will be included on my report to the authors and, if the manuscript is accepted for publication, my named report including any attachments I upload will be posted on the website along with the authors' responses. I agree for my report to be made available under an Open Access Creative Commons CC-BY license (http://creativecommons.org/licenses/by/4.0/). I understand that any comments which I do not wish to be included in my named report can be included as confidential comments to the editors, which will not be published.

Choose an item.

To further support our reviewers, we have joined with Publons, where you can gain additional credit to further highlight your hard work (see: https://publons.com/journal/530/gigascience). On publication of this paper, your review will be automatically added to Publons, you can then choose whether or not to claim your Publons credit. I understand this statement.

Yes Choose an item.
